# Neurotoxic amyloid β‐peptide and tau produce cytokine‐like effects on PMCA in glioblastoma cell lines, enhancing its activity and isoforms expression

**DOI:** 10.1002/2211-5463.70046

**Published:** 2025-05-05

**Authors:** María Berrocal, Alberto Alvarez‐Barrientos, Ana M. Mata

**Affiliations:** ^1^ Departamento de Bioquímica y Biología Molecular y Genética, Facultad de Ciencias Universidad de Extremadura Badajoz Spain; ^2^ Instituto de Biomarcadores de Patologías Moleculares (IBPM) Universidad de Extremadura Badajoz Spain; ^3^ Servicio de Técnicas Aplicadas a la Biociencia (STAB), Edificio Guadiana, SAIUEx Universidad de Extremadura Badajoz Spain

**Keywords:** astrocytes, Aβ, Ca^2+^‐ATPase, cytokines, PMCA, tau

## Abstract

The transformation of astrocytes into neurotoxic reactive astrocytes, classified as A1, by inflammatory cytokines, and their link to brain damage and neurodegenerative diseases has been widely documented. However, the roles of two biomarkers of Alzheimer's disease (AD), amyloid β‐peptide (Aβ) and tau, and that of calcium pumps which are involved in the fine‐tuning of calcium homeostasis, are poorly understood in astrocytes. In this study, we showed that treating astrocytoma U‐251 cells with a cocktail of cytokines significantly increased plasma membrane Ca^2+^‐ATPase (PMCA) activity and expression levels of the four PMCA isoforms. Moreover, treatment of cells with Aβ1‐42 or tau induced a similar upregulation of PMCA activity and isoform expression levels as cytokines. These effects support the close association of Aβ and tau with inflammation. This study may help better understand the role of PMCA in promoting calcium extrusion from astrocytes transformed by AD markers.

## Graphical abstract

Two biomarkers of Alzheimer's disease, amyloid β‐peptide (Aβ) and tau, induce the transformation of U‐251 and other glioblastoma cell lines into neurotoxic A1‐like reactive astrocytes. This transformation is produced by cytokines and is followed by upregulation of PMCA activity and isoform expression, and is closely associated with inflammation, as revealed by increased cell death and ROS generation.

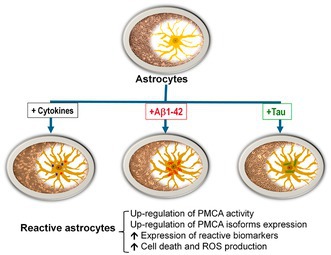

**Abbreviations**
AD: Alzheimer's disease
Aβ: amyloid β‐peptide
C1q: complement component 1q
CAV1: caveolin‐1
GFAP: glial fibrillary acidic protein
IL‐1α: interleukin‐1α
PMCA: plasma membrane Ca^2+^‐ATPase
TNF‐α: tumor necrosis factor‐α

The amyloid β‐peptide (Aβ) and tau protein are the main components of toxic amyloid plaques and aberrant phosphorylated tau tangles, two histopathological hallmarks of Alzheimer's disease (AD), a progressive and most frequent neurodegenerative disorder with high prevalence in the aging population. AD is a multifactorial disease, and one important factor involved in the disease is astrogliosis, which is characterized by an increase in reactive astrocytes with the progression of the disease. They are the major type of glial cells, which surround amyloid plaques in the AD human brain [1, 2] and AD‐transgenic mice [3], and contribute to the inflammatory response and calcium signaling dysregulation [4, 5, 6, 7, 8].

Reactive astrocytes could be classified into neurotoxic A1 and neuroprotective A2 types [9]. The A1 astrocytes are induced by activated neuroinflammatory microglia, or by the three cytokines secreted by activated microglia, interleukin‐1α (IL‐1α), tumor necrosis factor‐α (TNF‐α) and complement component 1q (C1q) *in vitro*, and they are found in many human neurodegenerative diseases [9]. Although astrocytes are nonexcitable cells, they show intrinsic Ca^2+^ fluctuations in the absence of external signals [10]. In fact, their activities are determined by intracellular Ca^2+^ signaling [11], which is involved in essential functions of the central nervous system, such as ion transport across plasma and intracellular cell membranes, synaptic transmission, cell plasticity, or behavior [12]. Besides, astrocyte Ca^2+^ activity has been shown to be upregulated by Aβ peptides [6, 13, 14], and located near amyloid plaques, as revealed in transgenic mouse models of AD [15]. There are controversial results about Ca^2+^ responses to Aβ in astrocytes, depending on the length and aggregation state of the peptide used and the period of treatments. Thus, some studies have reported that exposure of cultured astrocytes to Aβ1‐42 for short periods (5 min to 6 h) led to an increase in resting intracellular Ca^2+^ levels [6, 16], while others have shown that longer incubations with nm concentrations of the peptide for up to 48 h did not affect resting intracellular Ca^2+^ levels [17, 18]. In addition, the association of Aβ with Ca^2+^ dysregulation and inflammation has been widely documented. Thus, astrocytes treated with Aβ increased significantly the expression of glial fibrillary acidic protein (GFAP), a biomarker of A1‐like reactive astrocytes [9], due to endoplasmic reticulum Ca^2+^ release [19].

The treatment of primary astrocyte cultures with Aβ increases the production of inflammatory cytokines and induces neurotoxicity and tau phosphorylation [20], while the attenuation of astrocyte inflammation mitigates tau pathology [21]. On the contrary, GFAP‐positive astrocytes can internalize tau and may contribute to its propagation [22, 23]. Endogenous astrocytic tau is required for astrocyte‐mediated synaptotoxicity induced by Aβ and the accumulation of brain‐derived AD tau fibrils induces a more robust inflammatory and neurotoxic phenotype in human astrocytes, highlighting the nature of tau fibrils as an important contributing factor to inflammation and neurodegeneration in AD [24]. In line with that, it has been shown [25] that Aβ is associated with an increase in plasma phosphorylated tau only in individuals with astrocyte reactivity.

A key modulator of Ca^2+^‐dependent signaling is the plasma membrane Ca^2+^‐ATPase (PMCA) that releases the excess of cytosolic Ca^2+^ out of the cell at the expense of ATP hydrolysis. There are four main PMCA isoforms encoded by different genes and more than 30 additional variants generated by alternative splicing of each PMCA gene transcript [26, 27]. Isoforms differ in their tissue expression and distribution and in their role in cytosolic Ca^2+^ regulation [28, 29]. The PMCA has been studied in many cell types and species. However, only a few studies have reported the presence of PMCA in astrocytes [30, 31, 32, 33, 34]. Besides, the overexpression of the PMCA2w/b isoform in HEK‐293 cells leads to a significant reduction in the amplitude and duration of Ca^2+^ signals [33]. A similar reduction in Ca^2+^ signals was observed in Aβ‐preconditioned primary astrocytes isolated from mice [34] and it was proposed that this reduction was due to the potentiation of Ca^2+^ extrusion via PMCA and the activation of the cAMP signal. On the contrary, we have reported [35] that store‐operated calcium entry (SOCE) was inhibited after the transformation of U251 astrocytes into A1‐like astrocytes by cytokines treatment, and this results in a significant reduction of the Ca^2+^ stored in the ER. However, the cytosolic Ca^2+^ levels were not increased. Western blot experiments showed an increase in total PMCA expression in treated astrocytes. Taking those findings, we, then, suggested that the attenuation of Ca^2+^ entry through SOCE and the upregulation of PMCA expression could efficiently counterbalance the enhanced Ca^2+^ release from the ER in A1‐like astrocytes [35].

In line with those studies, in this work we have analyzed the PMCA activity and expression levels of each PMCA isoform in astrocytoma U‐251 cells treated with cytokines and also with Aβ and tau. This study has been supplemented with kinetic assays performed in other glioblastoma and neuronal cell lines. Our findings are summarized as follows: (a) treatment of U‐251 astrocytes by cytokines induces the upregulation of the Ca^2+^‐ATPase activity of PMCA, but not that of the intracellular SERCA pump; (b) the Aβ peptide and tau (two molecular markers of AD) produce the same effects on the PMCA activity and isoform expression as cytokines; (c) all treatments induce viability reduction and an increment in apoptosis and ROS production in U‐251 astrocytes associated with their transformation to toxic A1‐like reactive astrocytes.

## Materials and methods

### Reagents

The human astrocyte‐derived glioblastoma cell line U‐251 (ECACC; cat. no.: 09063001) was from Merck Sigma‐Aldrich (Madrid, Spain). The human glioblastoma cell line U‐138 (ATCC; cat. no.: HTB‐16) and the rat glioma C6 cell line (ATCC; cat. no.: CCL‐107) were from American Type Culture Collection (ATCC, Manassas, VA, USA). IL‐1α and TNF‐α were supplied by PeproTech®, Thermo Fisher Scientific (Madrid, Spain) and complement component C1q was from Bio‐Rad (Hercules, CA, USA). The Aβ1‐42 peptide was supplied by StabVida as a lyophilized powder. A stock solution of 4 mg·mL^−1^ was prepared by dissolving the peptide in 1% NH_4_OH and diluting it with 100 mm HEPES/KOH (pH 7.4). Full‐length tau 441 was obtained from Enzo Life Sciences (Farmingdale, NY, USA). The following antibodies were used: monoclonal anti‐PMCA (5F10) and anti‐PMCA4 (JA9) were from Invitrogen, Thermo Fisher Scientific (Madrid, Spain); anti‐Aβ (6E10) from Enzo Life Sciences; anti‐Tau5 and anti‐GFAP were from Sigma‐Aldrich; the anti‐IL‐1α (B‐7) from Santa Cruz Biotechnology (Heidelberg, Germany); and the anti‐SERCA (IID8) from Sigma‐Aldrich. Polyclonal antibodies anti‐PMCA1, anti‐PMCA2, anti‐PMCA3, and anti‐C3 (JF10‐30) were from Thermo Scientific; the caveolin‐1 (N‐20) and the TNF‐α (N‐19) antibodies were from Santa Cruz biotechnology. Fluorescently labeled secondary antibodies anti‐rabbit IgG‐Alexa488 and anti‐mouse IgG‐Alexa633 were from Invitrogen, Thermo Fisher Scientific. Thapsigargin was purchased from Sigma‐Aldrich.

### Cell cultures and treatments

Cell lines U‐251, U‐138, and C6 were grown in Dulbecco's Modified Eagle's Medium (DMEM) supplemented with 2 mm l‐glutamine, 100 mU·mL^−1^ penicillin, 0.1 mg·mL^−1^ streptomycin, and 10% heat‐inactivated fetal bovine serum (FBS). Cells were incubated at 37 °C in humidified atmospheric air containing 5% CO_2_. Reactive astrocytes (specified as A1, A1‐like, neuroinflammatory or neurotoxic astrocytes, [36]) were prepared by growing cells to 70% confluence and then replacing the culture medium with FBS‐free DMEM supplemented with glutamine and antibiotics. The next day, cells were stimulated for 24 h with a cocktail of pro‐inflammatory cytokines: IL‐1α (3 ng·mL^−1^), TNF‐α (30 ng·mL^−1^), and C1q (400 ng·mL^−1^), as described in [9] or alternatively with 5 μm Aβ1‐42 or 10 nm tau. Control astrocyte cells were always maintained in supplemented DMEM with FBS. Neuronal cell lines SH‐SY5Y, HT‐22, and N2a were grown and treated as described for astroglioma cell lines.

### Homogenates and membranes preparation

Cells without any treatment (control cells, C) or treated as previously described were harvested, washed twice with phosphate‐buffered saline (PBS), and centrifuged for 3 min at 1700 ***g***. The collected pellet was resuspended in 10 mm HEPES/KOH, pH 7.4, 0.32 m sucrose, 0.5 mm MgSO_4_, 0.1 mm PMSF, 2 mm 2‐mercaptoethanol, and protease inhibitor cocktail solution (Roche Diagnostics, Barcelona, Spain) by using the homogenizer Omni Tissue Master 125 with a 5‐mm flat bottom probe (Omni Int., Kennesaw GA, USA). The homogenate was then centrifuged for 1 min at 5000 ***g***, collecting the supernatant and stored at −80 °C. Cell membranes were prepared as described by Sepulveda *et al*. [37]. Briefly, cells were scraped and pelleted. The cell pellet was homogenized in 10 mm HEPES/KOH, pH 7.4, 0.32 m sucrose, 0.5 mm MgSO_4_, 0.1 mm PMSF, 2 mm 2‐mercaptoethanol, and protease inhibitor cocktail solution (Roche) and centrifuged for 10 min at 1500 ***g***, and the supernatant was centrifuged for 45 min at 100 000 ***g***. The pellet containing cell membranes was resuspended in 10 mm HEPES/KOH, pH 7.4, 0.32 m sucrose, and stored at −80 °C until use in aliquots. Protein concentration was determined by the Bradford method [38].

### Ca^2+^‐ATPase activity

The total Ca^2+^‐ATPase activity was measured by using a coupled enzyme assay, following the absorbance decrease in NADH at 340 nm, as described by Salvador and Mata [39]. Cell membranes (20 μg) were incubated for 2 min at 37 °C in 1 mL of assay medium containing 50 mm HEPES/KOH, pH 7.4, 100 mm KCl, 2 mm MgCl_2_, 5 mm NaN_3_, 0.22 mm NADH, 0.42 mm phosphoenolpyruvate, 10 IU pyruvate kinase (Roche, Ref.: 10109045001) and 28 IU lactate dehydrogenase (Roche; Ref.: 10127230001), 0.01% saponin, and the indicated free Ca^2+^ concentration, expressed as pCa, and adjusted by the appropriate addition of CaCl_2_ and BAPTA. After incubation, the reaction was triggered with 2 mm ATP followed by addition of 6 mm EGTA (to measure Mg^2+^‐ATPase activity). To determine SERCA and PMCA activities, the reaction was started with 2 mm ATP followed by successive additions of 100 nm thapsigargin (to inhibit SERCA activity) and 6 mm EGTA. The SERCA activity was determined by subtracting the activity value in the presence of thapsigargin from the total activity. The PMCA activity was calculated by subtracting the contribution of Mg^2+^‐ATPase activity from the activity value obtained in the presence of thapsigargin. Ca^2+^‐ATPase activities of neuronal cell lines were measured by using the same assay as for astroglioma cell lines, and 3.16 μm free calcium.

### Flow cytometry

U‐251 cells were seeded overnight at a density of 150 000 cells in 35‐mm cell culture plates and treated as described above. Afterward, cells were harvested using PBS and centrifuged for 3 min at 1700 ***g***. Then, cells were fixed with 3% paraformaldehyde (PFA), 3 mm MgCl_2_, 2 mm EGTA, and 0.32 m sucrose in PBS, pH 7.4, for 30 min at 37 °C with gentle shaking. Afterward, cells were centrifuged for 5 min at 4000 ***g*** to remove the PFA and washed three times with PBS and 0.1% bovine serum albumin (BSA). Fixed cells were permeabilized and blocked with 1% BSA in PBS supplemented with 0.2% Triton X‐100 (PBST) for 1 h at room temperature (RT) and washed three times with PBS (washing step). Cells were then incubated with the corresponding primary antibodies (anti‐PMCA1, anti‐PMCA2, anti‐PMCA3, anti‐PMCA4, 5F10, 6E10, and a‐Tau5) diluted 1 : 200 in PBST, overnight at 4 °C with gentle rotation. Afterward, cells were extensively washed with PBST and incubated for 2 h with the appropriate Alexa488‐labeled or Alexa633‐labeled secondary antibodies diluted 1 : 200 in PBST with rotation and protected from light, and washed with PBST. Cells‐stained analysis was performed on the Cytoflex S flow cytometer (Beckman Coulter, Inc. Miami, FL, USA), and controls with secondary antibodies were used to set up background. Forward scatter (FSC) was used as a threshold parameter, and singlets were identified by using FSC‐Area and FSC‐width dot plot. A 488‐nm laser was used to excite the Alexa 488 secondary antibody, and a 642‐nm laser was used to excite the Alexa 633 secondary antibodies. Fluorescence emission was detected by APDs with 525/40‐nm BP filter and 660/10‐nm BP filter. Fluorescence analysis was performed on cells gated by FSC/SSC to assure that cells with higher SSC and lower FSC (apoptotic) were not included. Fluorescence data are presented as mean fluorescence intensity (MFI), which is a measure of the distribution of the fluorescence intensity in each fluorescence detector, as a function of the channel resolution of the instrument. The CytExpert software (Beckman Coulter) was used for data analysis and representation.

### Dot blot assay

Homogenates (10 μg) from U‐251 cells were spotted on a sealed nitrocellulose membrane using a Bio Dot® SF apparatus (Bio‐Rad). Total control protein was detected by Ponceau S staining. After blocking in Tris‐buffered saline (TBS) containing 5% (w/v) low‐fat milk for 1 h at RT, immunodetections were performed by incubating the membranes with the primary antibodies anti‐GFAP (1 : 20 000) for 5 min at RT or with the anti‐C3 (1 : 500), anti‐CAV1 (1/200), anti‐IL‐1α (1 : 500) and anti‐TNF‐α (1 : 500), diluted in TBS‐0.05% (v/v)‐Tween 20, overnight at 4 °C. Afterward, membranes were incubated for 1 h at RT with peroxidase‐conjugated secondary antibodies (1 : 3000) and after washing, the ECL substrate (Bio‐Rad) was applied to the membranes and signals were visualized with a Chemidoc™ XRS+ Imaging System (Bio‐Rad) and quantified with image lab™ software 3.0.

### Cell viability and reactive oxygen species (ROS) detection

Both assays were performed by seeding U‐251 cells overnight at a density of 10 000 cells per well in 96‐well cell culture plates and then treated as previously described. Viable cells were detected by the colorimetric MTT assay, based on the reduction in the yellow tetrazolium MTT (3‐(4,5‐dimethylthiazol‐2‐yl)‐2,5‐diphenyltetrazolium bromide) by NAD(P)H‐dependent oxidoreductase enzymes of viable cells to purple formazan crystals that can be measured spectrophotometrically [40]. Briefly, control and treated cells were incubated with 200 μL of 150 μg·mL^−1^ MTT in PBS for 1 h in an incubator (37 °C, 5% CO_2_). Next, the MTT was removed and 100 μL of DMSO was added to each well to dissolve formazan crystals. Viable cells were quantified at 490 and 650 nm (background subtraction) in a Varioskan Flash fluorescence spectrophotometer (Thermo Scientific). Intracellular ROS production was measured in control and treated cells, with the fluorogenic dye 2′7′‐dichlorodihydrofluorescein diacetate (DCFH‐DA), according to [41]. Forty μm of DCFH‐DA in PBS was added to cells and incubated for 30 min in an incubator (37 °C, 5% CO_2_). The DCFH‐DA diffuses into cells and is deacetylated to a nonfluorescent compound that is then oxidized to the fluorescent product 2′7′‐dichlorofluorescein (DCF) by ROS. After washing with PBS, cells were lysed with 25 μL NaOH 1 m and PBS was added up to 200 μL final volume. The fluorescent intensity of cell extracts was measured using λexc = 485 nm and λem = 530 nm.

### Quantification of apoptotic cells

Apoptosis detection was evaluated using 4′6‐diamidino‐2′‐phenylindole dihydrochloride (DAPI) staining in U‐251 cells seeded overnight at a density of 60 000 per well in 24‐well plates and then treated as described above. For DAPI staining, cells were fixed and incubated with 0.3 μm of DAPI fluorescent dye to stain cell nuclei. Apoptotic cells showed condensed chromatin, and nonapoptotic cells presented a homogeneous distribution of DNA [42].

### Statistical analysis

Data were fitted to the appropriate equation using the sigmaplot v10 software (SPSS Inc, Chicago, IL, USA). Significant differences were determined by an unpaired Student's *t*‐test. Statistical significance was accepted for *P* ≤ 0.001.

## Results

This work shows the effects of a cocktail of cytokines (Il‐1α, TNF‐α, and C1q) and the AD biomarkers Aβ1‐42 and tau on the human astrocytic U‐251 cell line with respect to endogenous Ca^2+^‐ATPase activity and expression levels of specific PMCA isoforms.

**Fig. 1:**
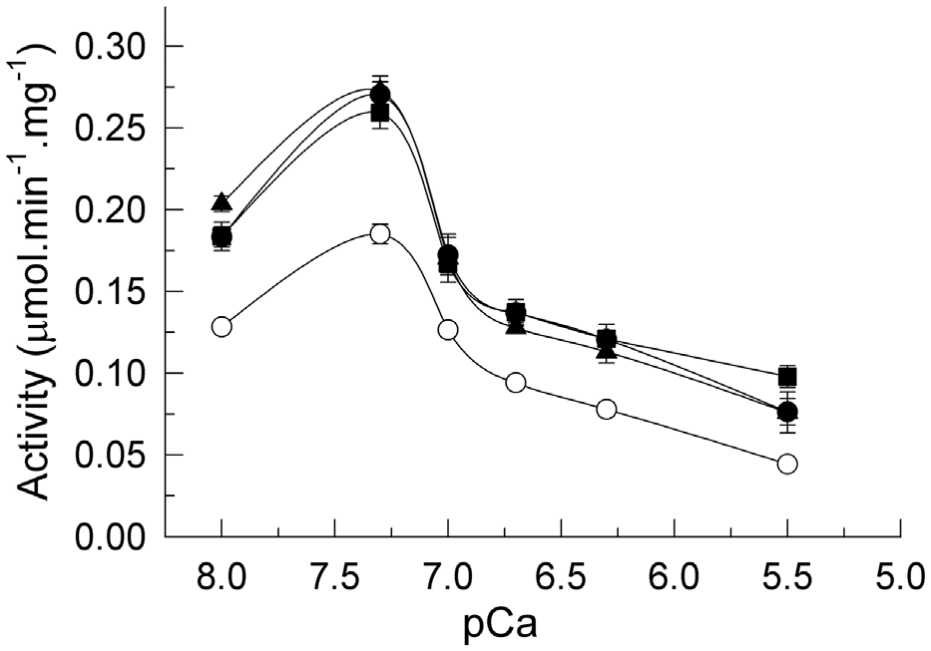
Ca^2+^ dependence of total Ca^2+^‐ATPase activity in membranes from U‐251 cells untreated (○) or treated with a cocktail of cytokines IL‐1α (3 ng·mL^−1^), TNF‐α (30 ng·mL^−1^), and C1q (400 ng·mL^−1^) (●), or with 5 μm Aβ (▲) or 10 nm tau (■). Total Ca^2+^‐ATPase activity was measured as described in the Materials and methods section. Values of pCa correspond to free Ca^2+^ concentrations used in the assay. Results represent means ± SE (bars) of three experiments performed in duplicate with three different membrane preparations.

We have prepared A1‐like reactive astrocytes by treating U‐251 cells with the cytokines TNF‐α (30 ng·mL^−1^), IL‐1α (3 ng·mL^−1^), and C1q (400 ng·mL^−1^) for 24 h, following, basically, the procedure described by [9]. As shown in previous work [35] this treatment induces reactivity in the U251 cell line. Alternatively, cells were treated with the amyloid peptide Aβ1‐42 (5 μm) or with tau (10 nm) to analyze whether these compounds also induce astrocytic reactivity. First, we performed the Ca^2+^‐ATPase activity assay in membranes prepared from untreated and treated U‐251 cells, using the same conditions that we have previously applied for brain samples or neuronal cultures [39, 43]. However, we did not obtain a significant activity. Therefore, we had to optimize the conditions of the activity assay in astrocytes with respect to the optimal concentration of free calcium. Figure 1 shows the effect of free calcium on the total Ca^2+^‐ATPase activity. As shown, the activity was very sensitive to Ca^2+^ concentrations, reaching the maximum value at 0.05 μm free Ca^2+^ (pCa of 7.3), independently of cell culture treatments. Besides, the Ca^2+^‐ATPase activity of astrocytes membranes treated with cytokines, Aβ or tau was upregulated (about 2‐fold) with respect to that of nontreated cells.

**Fig. 2:**
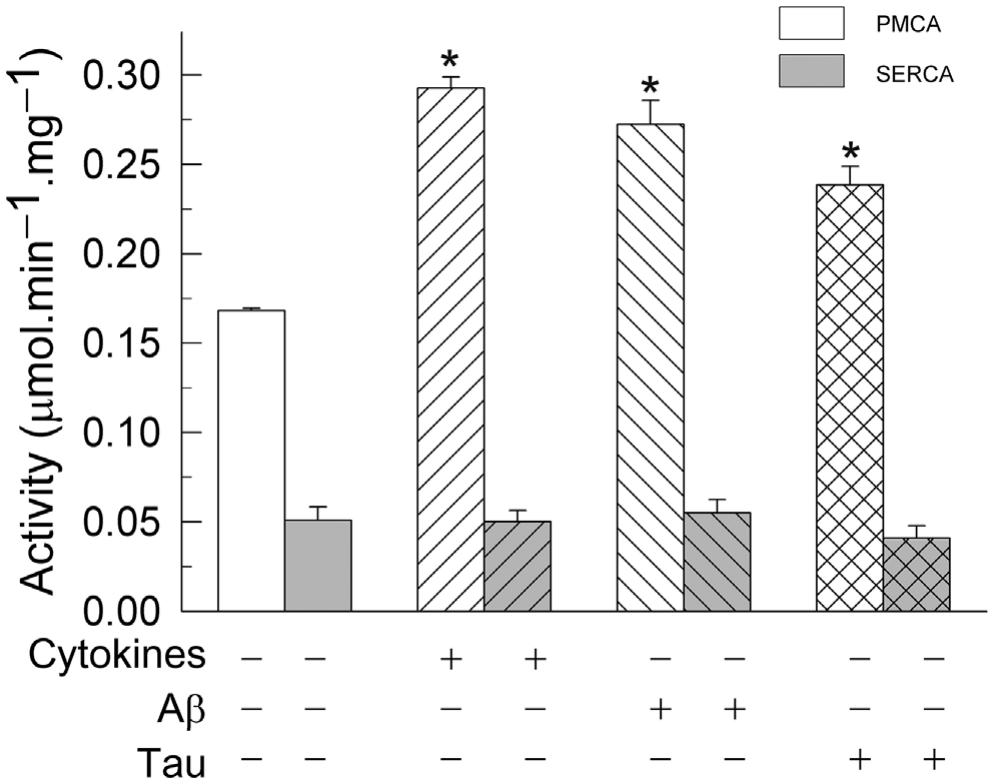
Treatments of U‐251 cells with a cocktail of cytokines (3 ng·mL^−1^ of IL‐1α, 30 ng·mL^−1^ of TNF‐α, and 400 ng·mL^−1^ of C1q) or with 5 μm Aβ or 10 nm tau upregulate the Ca^2+^‐ATPase activity of plasma membrane Ca^2+^‐ATPase (PMCA), but not that of SERCA pump. The Ca^2+^‐ATPase activity was measured in 20 μg of membranes from control and treated U‐251 cells, using 50 nm free Ca^2+^ and 100 nm thapsigargin to calculate the contribution of SERCA and PMCA to the total activity, as detailed in the Materials and methods section. Values represent mean ± SE from three experiments performed in duplicate and with three different preparations. Student's *t*‐test *P ≤ 0.001 vs. control.

To understand whether the activity upregulation is a common feature of calcium pumps from plasma and intracellular membranes, we analyzed the contribution of PMCA and SERCA activities to the total Ca^2+^‐ATPase activity at the optimum pCa of 7.3, in control and treated membranes, using the intracellular SERCA pump inhibitor thapsigargin. As shown in Fig. 2, only the PMCA activity was upregulated in astrocytoma cells treated with cytokines or Aβ or tau, while the SERCA activity was not affected by any of the treatments. This upregulation was also observed in rat glioblastoma C6 cells and in human U‐138 glioblastoma cells (Table 1).

**Table 1:** Ca^2+^‐ATPase activity in membranes from human glioblastoma (U‐251, U‐138) and rat glioma (C6) cell lines, untreated (Control) and treated with cytokines (3 ng·mL^−1^ of IL‐1α, 30 ng·mL^−1^ of TNF‐α, and 400 ng·mL^−1^ of C1q), or with 5 μm Aβ1‐42 or 10 nm tau. Values represent mean ± SE from 10 (or four) experiments performed in duplicate and with 10 preparations of U‐251 and four preparations of U‐138 and C6 cells, respectively. *P* values were obtained using Students *t*‐test.

Cell line	Treatment	Activity (μmol·min^−1^·mg^−1^)	*P* value
U‐251	Control	0.183 ± 0.003	
	Cytokines	0.304 ± 0.007	0.00001
	Aβ	0.296 ± 0.006	0.00001
	Tau	0.279 ± 0.005	0.00001
U‐138	Control	0.226 ± 0.004	
	Cytokines	0.364 ± 0.01	0.0007
	Aβ	0.359 ± 0.02	0.0016
	Tau	0.347 ± 0.01	0.0010
C6	Control	0.160 ± 0.004	
	Cytokines	0.290 ± 0.01	0.0001
	Aβ	0.272 ± 0.005	0.0001
	Tau	0.270 ± 0.02	0.003

**Fig. 3:**
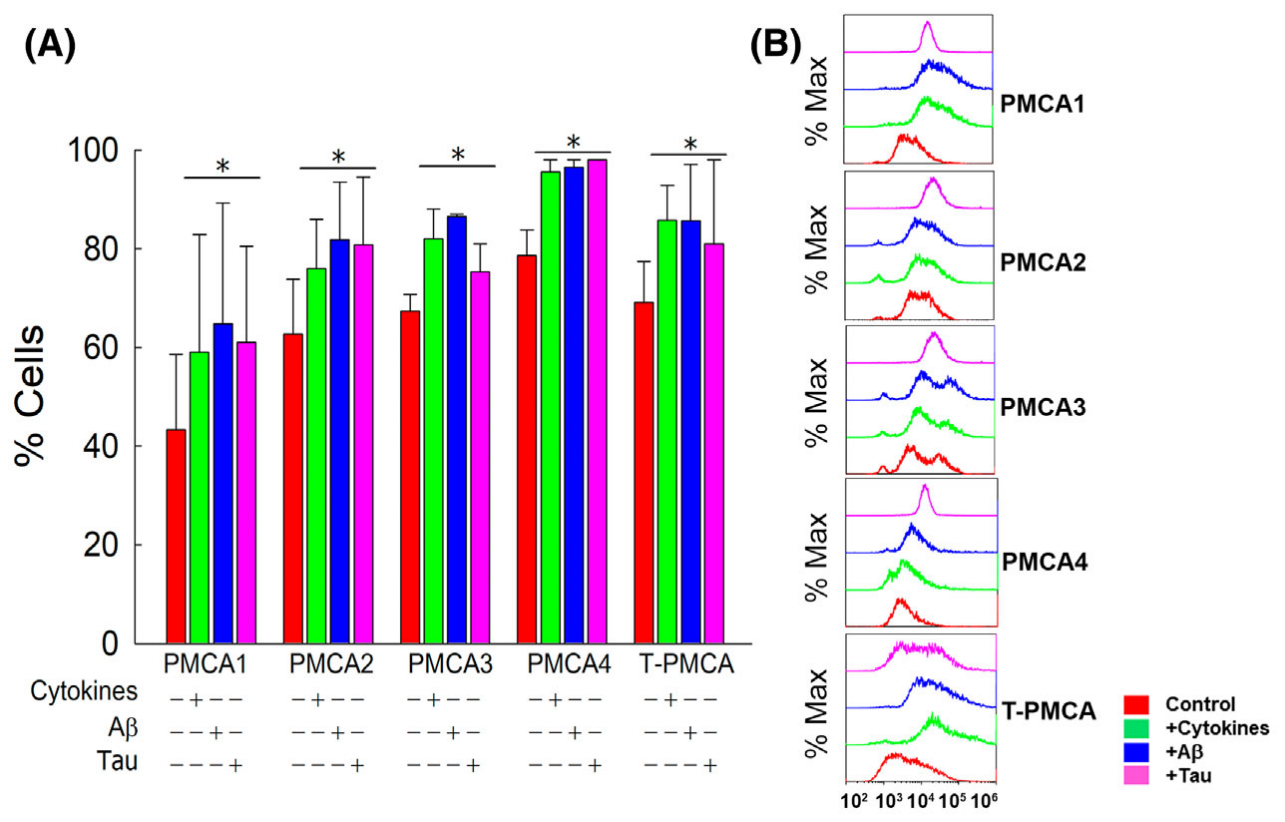
(A) Flow cytometry analysis shows that U‐251 cells express the four plasma membrane Ca^2+^‐ATPase (PMCA) isoforms. Besides, the percentage of cells expressing each isoform increases after their treatment with cytokines (3 ng·mL^−1^ of IL‐1α, 30 ng·mL^−1^ of TNF‐α, and 400 ng·mL^−1^ of C1q), 5 μm Aβ1‐42 or 10 nm tau, with respect to untreated cells. T‐PMCA (total PMCA) represents data obtained with the 5F10 antibody, that recognizes all PMCA isoforms. Control and treated cells were grown, fixed, permeabilized, blocked and incubated with specific primary and Alexa Fluor secondary antibodies as described in the Materials and methods section. Data are mean ± SE of eight experiments performed with eight different preparations. Student's *t*‐test *P ≤ 0.001 vs. control. (B) Overlay of flow cytometry histograms of different stainings and treatments from one representative experiment.

**Fig. 4:**
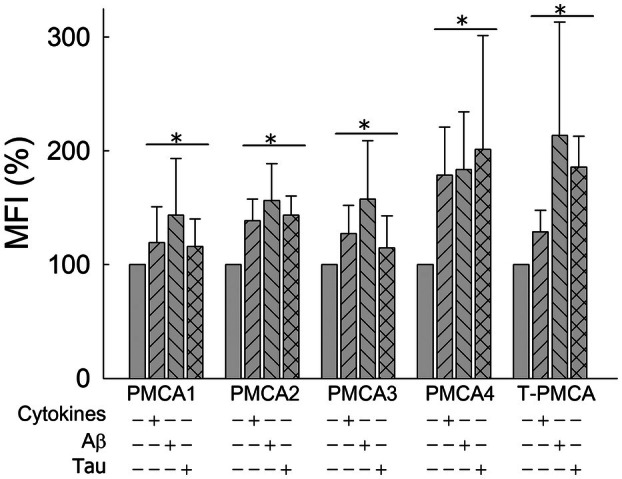
Flow cytometry data, presented as mean fluorescence intensity (MFI), show that percentages of each isoform expressed in individual U‐251 cells increase after their treatments with cytokines (3 ng·mL^−1^ of IL‐1α, 30 ng·mL^−1^ of TNF‐α, and 400 ng·mL^−1^ of C1q), 5 μm Aβ1‐42 or 10 nm tau with respect to untreated cells. T‐PMCA (total PMCA) represents data obtained with the 5F10 antibody that recognizes all PMCA isoforms. Control and treated cells were grown, fixed, permeabilized, blocked and incubated with specific primary and Alexa Fluor secondary antibodies as described in the Materials and methods section. Data are mean ± SE of eight experiments performed with eight different preparations. Student's *t*‐test *P ≤ 0.001 vs. control. PMCA, plasma membrane Ca^2+^‐ATPase.

The upregulation of Ca^2+^‐ATPase activity found in astrocytic cells after all treatments could be related to a rise in PMCA expression levels. In a previous study, we showed that PMCA expression was increased in astrocytes treated with cytokines [35]. In the present study, we analyzed in more detail the expression of PMCA, focusing on the four PMCA isoforms, in the astrocytic U‐251 cell line, before (control cells) and after being treated not only with cytokines but also with Aβ1‐42 or tau. The study was performed on individual human U‐251 astrocytic cells, using flow cytometry and specific PMCA‐isoform antibodies tagged to fluorochrome‐labeled secondary antibodies. As shown in Fig. 3A,B, each isoform was detected by flow cytometry in astrocytic cells. Besides, an upregulation of each isoform was observed in the cell ensemble after being treated with cytokines. Similar upregulations were observed in cells treated with 5 μm Aβ or 10 nm tau for 24 h. Also, the 5F10 antibody, which binds to the region of residues 719–738, highly conserved in the four PMCA isoforms, revealed 75% expression of total PMCA (T‐PMCA) in control cells and about a 25% increase in PMCA expression in cells treated with cytokines, Aβ or tau. In addition, flow cytometry data also indicated the relative amount of each isoform expressed by a single cell, based on the variation of MFI, meaning that an increase in MFI corresponds to higher antibody binding due to an increase in protein expression. Figure 4 shows an increase in fluorescence intensity (and thus, presumably expression) on each cell after treating the astrocytes with cytokines or Aβ or tau.

**Fig. 5:**
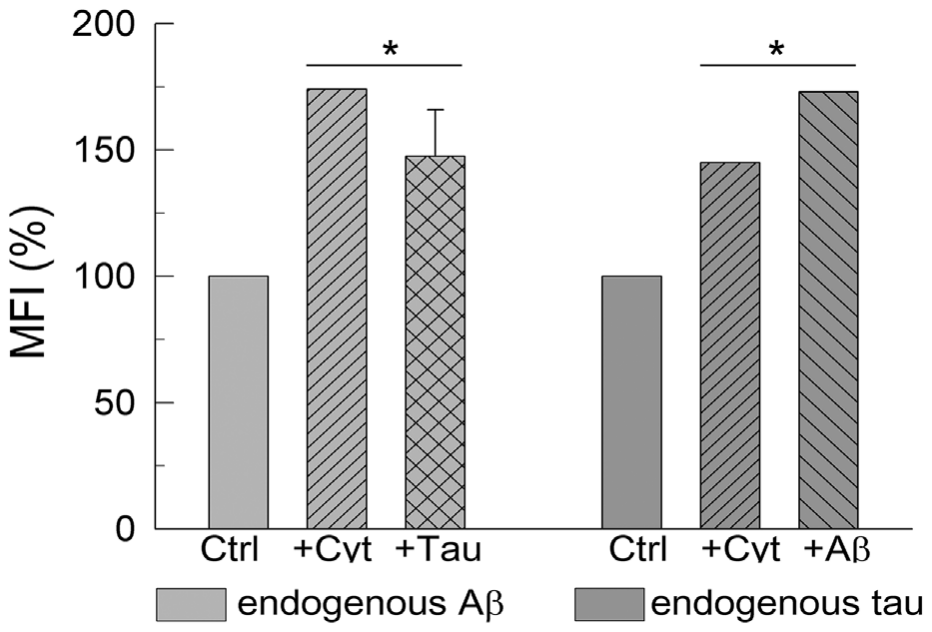
Flow cytometry analysis (presented as mean fluorescence intensity, MFI) of endogenous Aβ and tau in U‐251 untreated cells (Ctrl) or cells treated with cytokines (3 ng·mL^−1^ of IL‐1α, 30 ng·mL^−1^ of TNF‐α, and 400 ng·mL^−1^ of C1q), 5 μm Aβ1‐42 or 10 nm tau. These treatments significantly increase the MFI of endogenous Aβ and tau. Control and treated cells were grown, fixed, permeabilized and incubated with specific primary and fluorescent‐labeled secondary antibodies as described in the Materials and methods section. Data are mean ± SE of three experiments performed with three different preparations. Student's *t*‐test *P ≤ 0.001 vs. control.

We also used antibodies against Aβ1‐42 and tau (detected by fluorochrome‐labeled secondary antibodies) to visualize, by flow cytometry, endogenous levels of Aβ and tau in the astrocytic cells before and after treatments. The quantification of the MFI, relative to the expression of Aβ and tau on each cell (Fig. 5), shows that cells treated with cytokines or tau contained a higher expression of endogenous Aβ than control cells. Similarly, astrocytic cells treated with cytokines or Aβ showed a higher expression of endogenous tau than untreated cells.

**Fig. 6:**
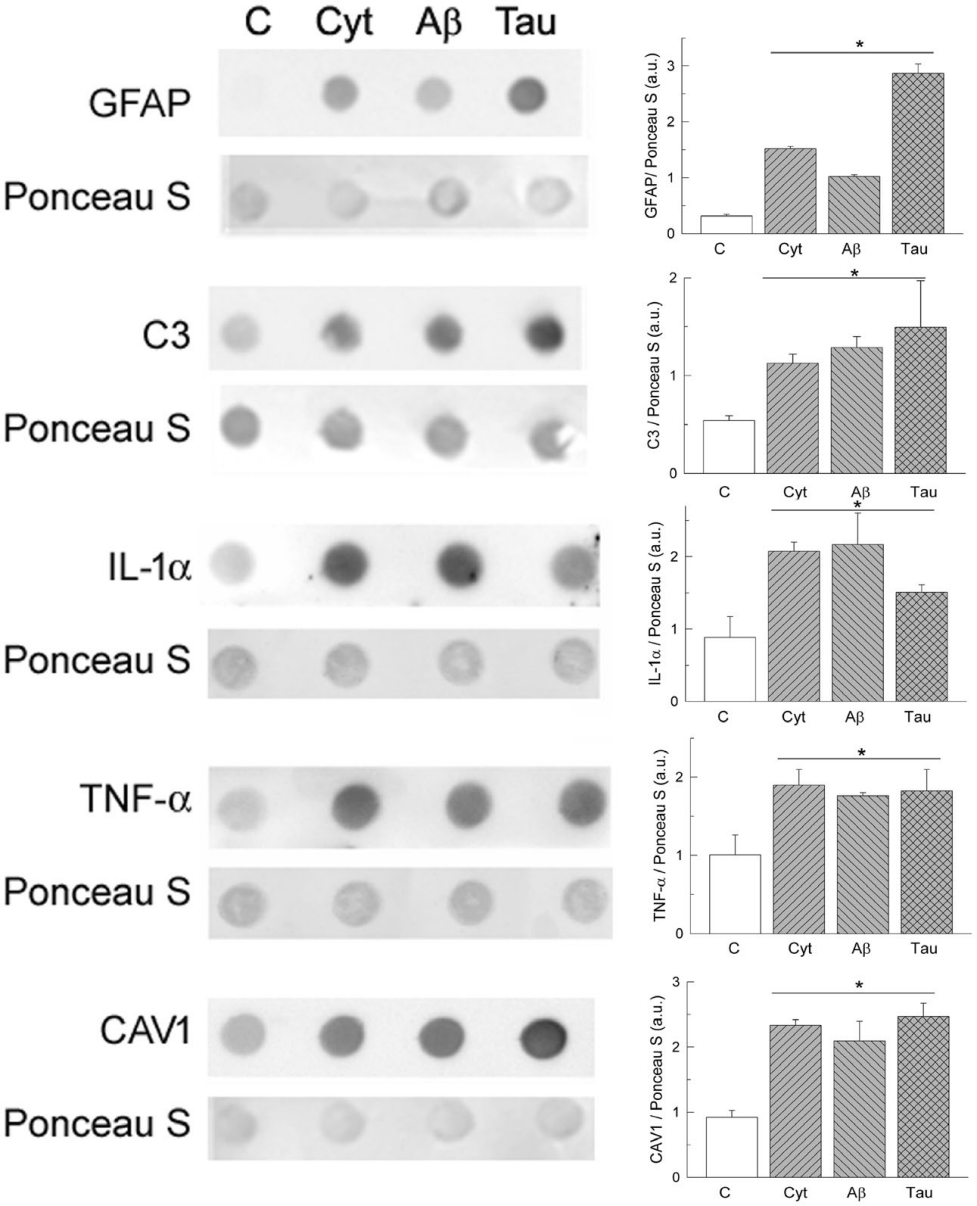
Expression of biomarkers of astrocyte reactivity, such as GFAP, C3, cytokines IL‐1a and TNF‐α, and CAV1 increases in U‐251 cells after been treated with cytokines (3 ng·mL^−1^ of IL‐1α, 30 ng·mL^−1^ of TNF‐α, and 400 ng·mL^−1^ of C1q), 5 μm Aβ1‐42 or 10 nm tau. Dot blots were performed with specific antibodies in cell homogenates, as described in the Materials and methods section. Staining with Ponceau S was used as protein loading control to quantify expression levels of each biomarker. Images show representative blots, and histograms show mean values ± SE from four experiments performed in duplicate with four different preparations. Student's *t*‐test *P ≤ 0.001 vs. control.

These results point out a close relationship between upregulation of PMCA activity, PMCA expression, and astrocyte reactivity. In regard to that, we assessed the expression of reactivity biomarkers in control and treated U‐251 cells, using dot blot immunoassays. Figure 6 shows that treatments of cells with cytokines, Aβ or tau significantly increased the expression of GFAP, a common molecular marker of astrocyte reactivity [3, 9]. In addition, the expression of C3 protein, a marker of neurotoxic reactive astrocytes [9, 24, 44], and pro‐inflammatory molecules, such as IL‐1α and TNF‐α [45], were also upregulated in cells treated with Aβ or tau. We also tested the expression of caveolin‐1 (CAV1), a cholesterol‐binding protein that is involved in the formation of caveolae [46], and is expressed in astrocytes and astroglioma cells [47], and upregulated in reactive astrocytes [48, 49]. Quantification of CAV1 by dot blot revealed higher levels of this protein in astrocytic cultures treated with cytokines, Aβ or tau compared with those found in control cultures.

**Fig. 7:**
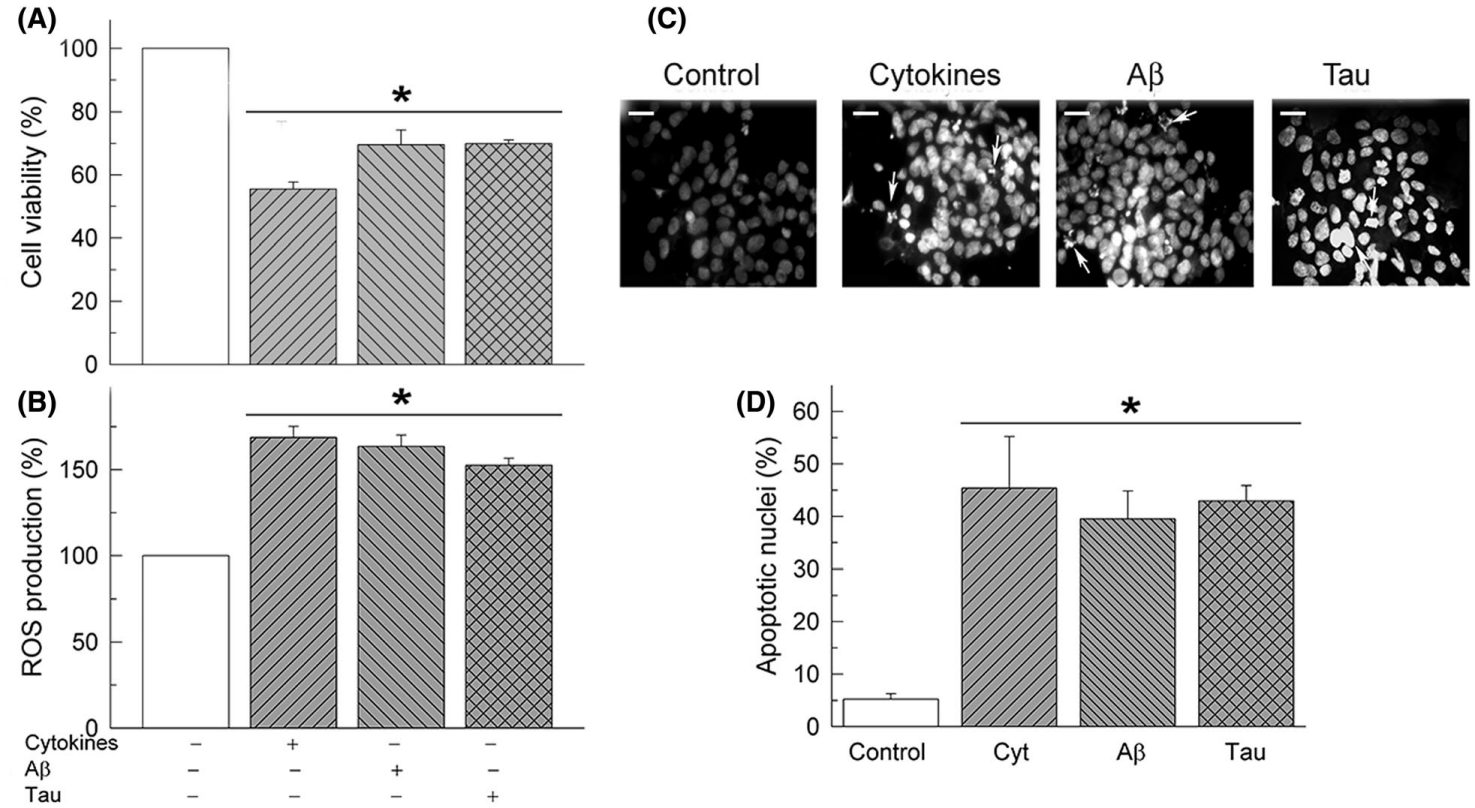
Treatments of U‐251 cells with cytokines, Aβ or tau reduce the percentage of viable cells (A) and increase the production of ROS (B) and the number of apoptotic cells (C, D). U‐251 cell cultures were treated without or with a cocktail of pro‐inflammatory cytokines, or with 5 μm Aβ1‐42 or 10 nm tau for 24 h, and then cell viability, ROS generation and apoptosis were assayed as described in the Materials and methods section. Data are expressed as percentage of viable cells and ROS, with respect to untreated cells, as mean ± SE of five independent experiments. Representative fluorescent microscopy images show apoptotic cells with condensed and fragmented nuclei (white arrows). Scale bar: 10 μm. Apoptotic nuclei were quantified with respect to the total number of seeded cells. Values are mean ± SE of 10 images per coverslip, obtained from four cell cultures. Student's *t*‐test *P ≤ 0.001 vs. control.

We and others have previously reported the cytotoxicity of Aβ and tau [43, 50, 51] in neuroblastoma cultures SH‐SY5Y. In this work, we have analyzed the effects of cytokines, Aβ or tau in the viability and oxidative stress in the U‐251 astrocytic cells. As can be seen in Fig. 7, all treatments reduced cell viability to 30–45% and increased mitochondrial ROS production by 50–70% compared with untreated cells. Furthermore, we visualized and quantified apoptotic nuclei with DAPI. Data revealed that about 45% of cells died after any of the three treatments, compared with 5% of dead cells observed in untreated cultures.

## Discussion

We have addressed the effects of inflammatory cytokines and the two AD molecular markers (Aβ and tau) on the Ca^2+^‐ATPase activity, and on the expression of specific PMCA isoforms, in the human glioblastoma U‐251 cell line, and their associations with reactive astrogliosis, cell viability, and ROS. A major finding of our work is the upregulation of the Ca^2+^‐ATPase activity found in these astrocytoma cells treated not only with cytokines but also with Aβ or tau (Fig. 1). However, only the plasma membrane PMCA pump showed a higher activity in treated cells than in control cells, while the activity of the intracellular SERCA was not affected by the cell treatments (Fig. 2). Similar effects were also observed in the other two glioma cell lines (human U‐138 and rat C6) tested (Table 1, Fig. S1). Therefore, it seems that the specific upregulation of PMCA activity by cytokines, Aβ and tau is a common event in astrocyte cells.

Besides, and independently of the treatments, the maximum value of total Ca^2+^‐ATPase activity in the astrocytic U‐251 cell line was obtained at 0.05 μm (pCa 7.3) free calcium (Fig. 1), while in neuroblastoma cultures, such as SH‐SY5Y (Fig. S2), membranes from human brain tissue and synaptosomes or purified PMCA from pig cerebrum [52], the *V*_max_ was reached at 3.16 μm (pCa 5.5) free calcium. These differences suggest that the PMCA pump has a higher affinity for Ca^2+^ in astrocytes than in neurons, and then, astrocyte PMCA is more active at lower Ca^2+^ concentrations than neuronal PMCA and will be able to maintain lower free cytosolic Ca^2+^ levels in astrocytic cells than in neuronal cells.

The stimulation of PMCA activity observed in astrocyte cell lines after being treated with Aβ or tau differs from the downregulation of PMCA activity (but not SERCA) found in our previous studies with SH‐SY5Y human neuroblastoma cells treated with Aβ [51] or with tau [50, 51], and it was supported in this work by treating this cell line and other neuronal cell lines, such as HT‐22 and N2a, with cytokines, Aβ or tau (Table S1). These differences could be explained by the modulation of different signaling molecules induced by the influx of cytokines, Aβ or tau in astrocytes and neurons.

In a recent study [35], we found that U‐251 cells treated with the three cytokines were transformed into A1‐like reactive astrocytes and displayed a significant reduction in cytosolic Ca^2+^ with respect to untreated cells, after depletion of intracellular Ca^2+^ stores mediated by thapsigargin (a specific inhibitor of SERCA) and in the presence of EGTA. Besides, the further addition of Ca^2+^ did not produce any increase in cytosolic Ca^2+^, compared with the large Ca^2+^ increase observed in untreated cells. This last effect was explained by inhibition of store‐operated calcium entry (SOCE), a major system of extracellular calcium entry. The low free cytosolic Ca^2+^ levels reported in that work after depletion of calcium stores by thapsigargin point to a system involved in the rapid release of Ca^2+^ out of the cells in A1‐like reactive astrocytes. The observations described in the present work about the upregulation of PMCA activity by cytokines and the lack of effect on SERCA strongly suggest that PMCA upregulation in A1‐like reactive astrocytes is responsible for the greater capacity of these astrocytes to release the cytosolic calcium out of the cell, compared with control astrocytes.

As mentioned above, the upregulation of PMCA, but not of SERCA activities, was also observed in astrocytes treated with Aβ1‐42. Using a different approach, Pham *et al*. [34] analyzed, by fluorescence microscopy, Ca^2+^ responses to Aβ25–35 stimulation in control and Aβ‐pretreated astrocytes (for 2 h) from hAPPJ20 AD mouse cortex. They showed that Aβ25‐35 stimulation induced a Ca^2+^ decrease in Aβ‐preconditioned astrocytes, and they proposed that this was due to a potentiation of Ca^2+^ extrusion by PMCA, among other molecular targets, because this effect was not observed in the presence of Aβ and PMCA inhibitors. In our work, we have used a kinetic NADH‐ATP‐coupled enzyme assay for Ca^2+^‐dependent ATPase activity, based on the quantification of the rate of ATP hydrolysis by the ATPase due to Ca^2+^ bound to the high affinity site of the enzyme and transported out of the cell. The assay was done in the absence and presence of thapsigargin, a specific noncompetitive SERCA inhibitor, to demonstrate that only the PMCA pump is specifically activated in the U‐251 glioblastoma cell line of human origin and other glioblastoma cell lines, such as the human U‐138 and rat C6, previously treated with 5 μm of Aβ1‐42. Our study also shows that the PMCA pump is upregulated in tau‐treated astrocytes. As far as we know, this effect has not been reported before. Furthermore, the activating effects produced by Aβ and tau on PMCA were like those found on astrocytes treated with cytokines.

The upregulation of PMCA in treated astrocytes could be linked to an increase in PMCA expression after those treatments, which could also be related to a reduction of cytosolic Ca^2+^ in astrocytes. The relationship between the increase in PMCA expression and cytosolic Ca^2+^ reduction has been suggested by [33], using hPMCA2w/b constructs to reveal astrocyte Ca^2+^ signaling *in vivo*. They found that hPMCA2w/b significantly reduced astrocyte basal Ca^2+^ levels (Ca^2+^ signals) and the effect was associated with an increase in self‐grooming in hPMCA2w/b‐expressing mice, although it did not have any effects on fast synapses and did not produce astrogliosis.

Flow cytometric analysis of PMCA expression on U‐251 cells revealed the presence of the four PMCA isoforms in these cells and that pretreatments of these astrocytes with cytokines, Aβ or tau reflect an upregulation of each isoform in the cell ensemble (Figs 3 and 4). Therefore, the upregulation of PMCA activity by cytokines, Aβ or tau seems to be closely associated with their higher expression on U251 astrocytes, induced by those treatments.

Previous immunoassays of protein expression, by dot blots, only gave a significant signal for the PMCA4 isoform (Fig. S3A), confirming its higher expression in cultures treated with cytokines, Aβ or tau, with respect to nontreated astrocytes. We may assume that these significant differences could depend on the type of technique used to detect the binding of antibodies to their epitopes on each specific isoform in astrocytes. The epitope exposure of each isoform to the antibody used to detect it may be different depending on cells treatment. Thus, the antibody could detect its epitope on a specific isoform in fixed and permeabilized astrocytic cells and may exhibit very poor binding to the isoform after sample treatment for western blots analysis. The sensitivity of detection is dependent on the exposure of the isoform epitope. In fact, [31] showed the presence of PMCA1,2 and 4 isoforms in rat cortical astrocytes, using western blots and RT‐PCR, and they also showed the presence of PMCA1 and 4 in C6 glioma cells by western blot. However, it has also been reported by RNA‐Seq that PMCA2, PMCA3, and PMCA4 are unlikely expressed in sorted striatal astrocytes at P30 by quantitative PCR, in comparison with PMCA1 [33]. Dot blots of SERCA also showed that the expression of this intracellular pump was not altered by any of the treatments (Fig. S3B).

Moreover, flow cytometry assays (Fig. 5) showed an increase in endogenous Aβ in cell cultures treated with cytokines or tau and an increase of endogenous tau in cultures treated with cytokines or Aβ, versus control astrocytes. These findings lead us to suggest that Aβ and tau induce astrocytic reactivity, associated with inflammation, like that generated by cytokines. This was proved by dot blot assays (Fig. 6), which showed a higher expression of biomarkers of astrocytic reactivity, such as GFAP, C3, pro‐inflammatory IL‐1α and TNF‐α, and CAV1, in astrocytes treated with Aβ or tau, with respect to control astrocytes, and similar to that detected in cytokine‐treated astrocytes. These observations confirmed that not only cytokines, but also Aβ and tau are able to induce the transformation of the astrocytic U‐251 cell line and other astroglioma cells into reactive A1‐like astrocytes.

We have recently shown the enhanced expression of endogenous Aβ in U‐251 cells treated with cytokines, using fluorescence measurements and dot blot assays [35]. Besides, other works have described that pro‐inflammatory cytokines are able to upregulate the amyloid‐beta precursor protein (APP) in human primary astrocytes [53, 54], leading to Aβ secretion. The close relationship between reactive astrocytes, inflammation, and Aβ pathology has been widely documented [55, 56]. As in microglia, astrocytes exposed to Aβ can release cytotoxic molecules which induce a neuroinflammatory response [57]. Astrocytes can internalize Aβ1‐42 very efficiently due to their phagocytic activity, and fibrillar Aβ increased the level of GFAP in human astrocytes [58]. Other works have reported that Aβ peptide induces an inflammatory response in primary astrocyte cultures from rats [59], and that reactive (GFAP‐positive) astrocytes, with high Aβ content, are often accumulated in AD‐affected brains [4, 5, 56, 60, 61], because they may reduce the capacity of astrocytes to degrade the peptide, and then, this can be a central process in the Aβ toxicity.

Endogenous tau is also expressed in astrocytes, as firstly reported by [62] in rats, and more recently in mice [63, 64] and humans [44, 65, 66, 67, 68], although at much lower levels than in neurons [63, 69]. Besides, it has been found that astrocytes can internalize tau from the extracellular medium through different mechanisms [70], and that astrocytic accumulation of tau fibrils in AD brains induces an inflammatory response in human astrocytes linked to AD‐neurodegeneration [71]. Astrocytic tau expression increased with aging and progression of AD and seems to be a required factor in the synaptotoxicity induced by Aβ [24, 25]. Although neuroinflammation has been frequently associated with a response induced by Aβ in AD, there are few studies showing that tau may play a significant role in neuroinflammation associated with neurodegeneration [20, 72]. Nilson *et al*. [73] have shown that tau oligomers co‐localize with astrocytes, microglia, and the high motility group box 1 (HMGB1) pro‐inflammatory cytokine, suggesting that tau oligomers may play a role in stimulating inflammation, although the molecular mechanism is far from clear. Another study [44] has shown that 4R tau promotes the neurotoxic reactive astrocytes characterized by the upregulation of C3 levels.

In contrast to the activating effect of cytokines, Aβ and tau on the function of endogenous PMCA, these treatments are neurotoxic for the cells, under the conditions used in this work, as revealed by a significant decrease in astrocytic cell viability, associated with an increase in ROS production and the increase in apoptotic cells (Fig. 7). However, the cells that remain alive after treatments show a high PMCA activity.

**Fig. 8:**
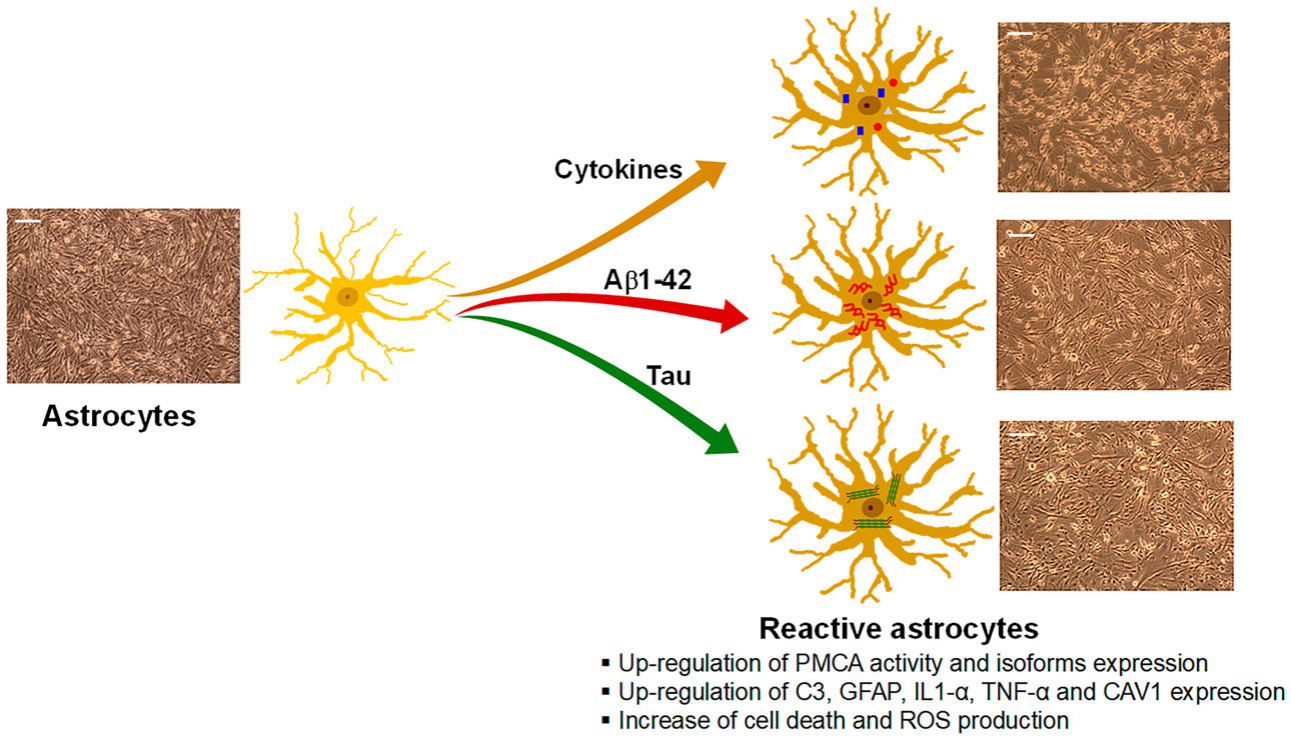
Scheme summarizing the effects of several treatments on astrocytoma cell lines.

In conclusion, this work (summarized in Fig. 8) reveals that endogenous PMCA is upregulated, and expression levels of PMCA isoforms are increased in astroglioma cell lines in response to cytokines and to Aβ1‐42 or tau. Besides, all treatments induce a significant increase in biomarkers of astrocyte reactivity associated with inflammation, cell death, and ROS production. These findings suggest that the AD molecular markers raise inflammatory responses like those produced by cytokines and stimulate the conversion of astrocytes to A1‐like reactive astrocytes. It seems that the activation of PMCA in reactive astrocytes may be a protective mechanism against the toxic effects of cytokines, Aβ or tau in calcium signaling.

## Conflict of interest

The authors declare no conflict of interest.

## Author contributions

MB designed and conducted experimental work, analyzed data, and contributed to writing, review, and editing. AB‐B provided expertise in flow cytometry assays, analysis of data, and editing. AMM planned the project, designed experiments, provided supervision, formal analysis, and interpretation, wrote the manuscript, review, and editing, and acquired funding. All the authors read and approved the final version of the manuscript submitted for publication.

## Supporting information


**Fig. S1.** Treatments of C6 glioma cells with Cytokines (3 ng·mL^−1^ of IL‐1α, 30 ng·mL^−1^ of TNF‐α, and 400 ng·mL^−1^ of C1q), or with 5 μm Aβ1‐42 or 10 nm tau upregulate the Ca^2+^‐ATPase activity of PMCA, but not that of SERCA pump.

**Fig. S2.** Ca^2+^ dependence of total Ca^2+^‐ATPase activity in membranes from human neuroblastoma SH‐SY5Y cells, untreated or treated with a cocktail of cytokines IL‐1α (3 ng·mL^−1^), TNF‐α (30 ng·mL^−1^), and C1q (400 ng·mL^−1^), or with 5 μm Aβ1‐42 or 10 nm tau.

**Fig. S3.** Treatment of U‐251 cells with a mixture of cytokines (3 ng·mL^−1^ of IL‐1α, 30 ng·mL^−1^ of TNF‐α, and 400 ng·mL^−1^ of C1q), or with 5 μm Aβ1‐42 or 10 nm tau, upregulates the expression of PMCA4, but does not affect SERCA expression.

**Table S1.** Downregulation of Ca^2+^‐ATPase activity in neuronal cell lines, after been treated with cytokines (3 ng·mL^−1^ of IL‐1α, 30 ng·mL^−1^ of TNF‐α, and 400 ng·mL^−1^ of C1q), or with 5 μm Aβ1‐42 or 10 nm tau.

## Data Availability

Data will be made available upon request.
