# Supplementary figures and images for: Neurotoxic amyloid β‐peptide and tau produce cytokine‐like effects on PMCA in glioblastoma cell lines, enhancing its activity and isoforms expression
Source: FEBS Open Bio. 2025 May 5;15(8):1350–64. doi: 10.1002/2211-5463.70046 (PMC12319713; doi:10.1002/2211-5463.70046)

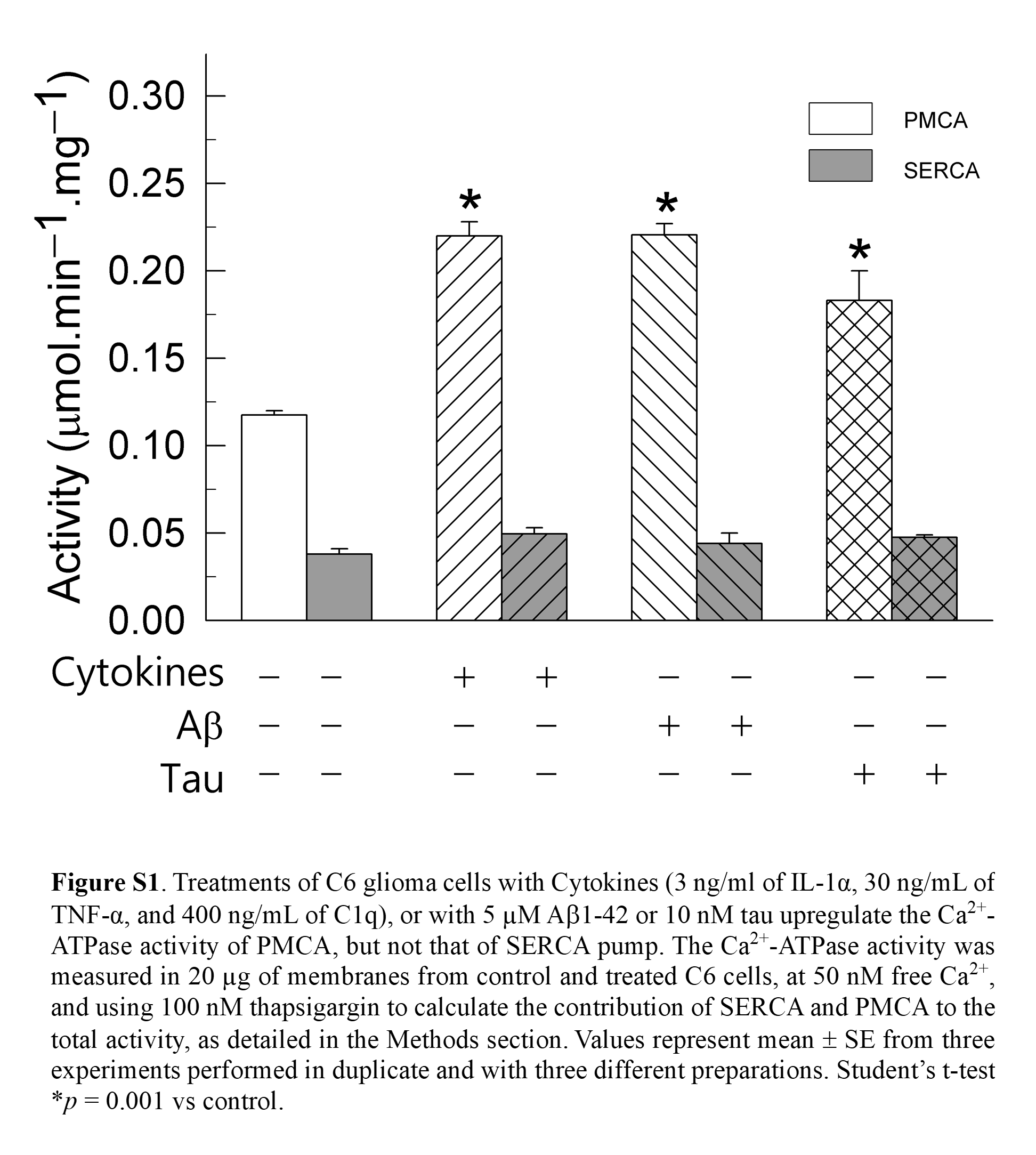

Supplement: Supplementary file 1 — Fig. S1. Treatments of C6 glioma cells with Cytokines (3 ng·mL−1 of IL‐1α, 30 ng·mL−1 of TNF‐α, and 400 ng·mL−1 of C1q), or with 5 μm Aβ1‐42 or 10 nm tau upregulate the Ca2+‐ATPase activity of PMCA, but not that of SERCA pump. [file FEB4-15-1350-s003.tif]

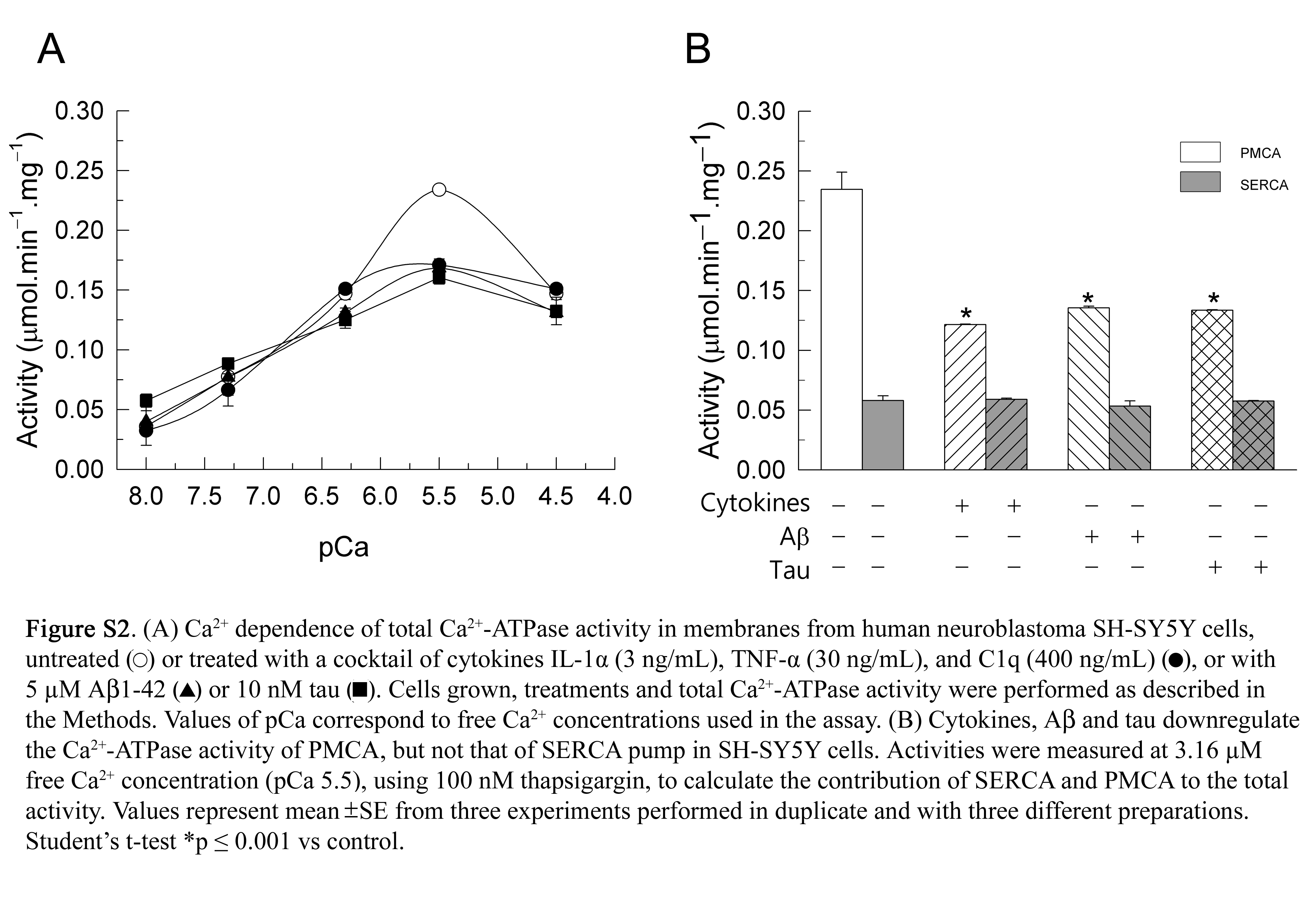

Supplement: Supplementary file 2 — Fig. S2. Ca2+ dependence of total Ca2+‐ATPase activity in membranes from human neuroblastoma SH‐SY5Y cells, untreated or treated with a cocktail of cytokines IL‐1α (3 ng·mL−1), TNF‐α (30 ng·mL−1), and C1q (400 ng·mL−1), or with 5 μm Aβ1‐42 or 10 nm tau. [file FEB4-15-1350-s001.tif]

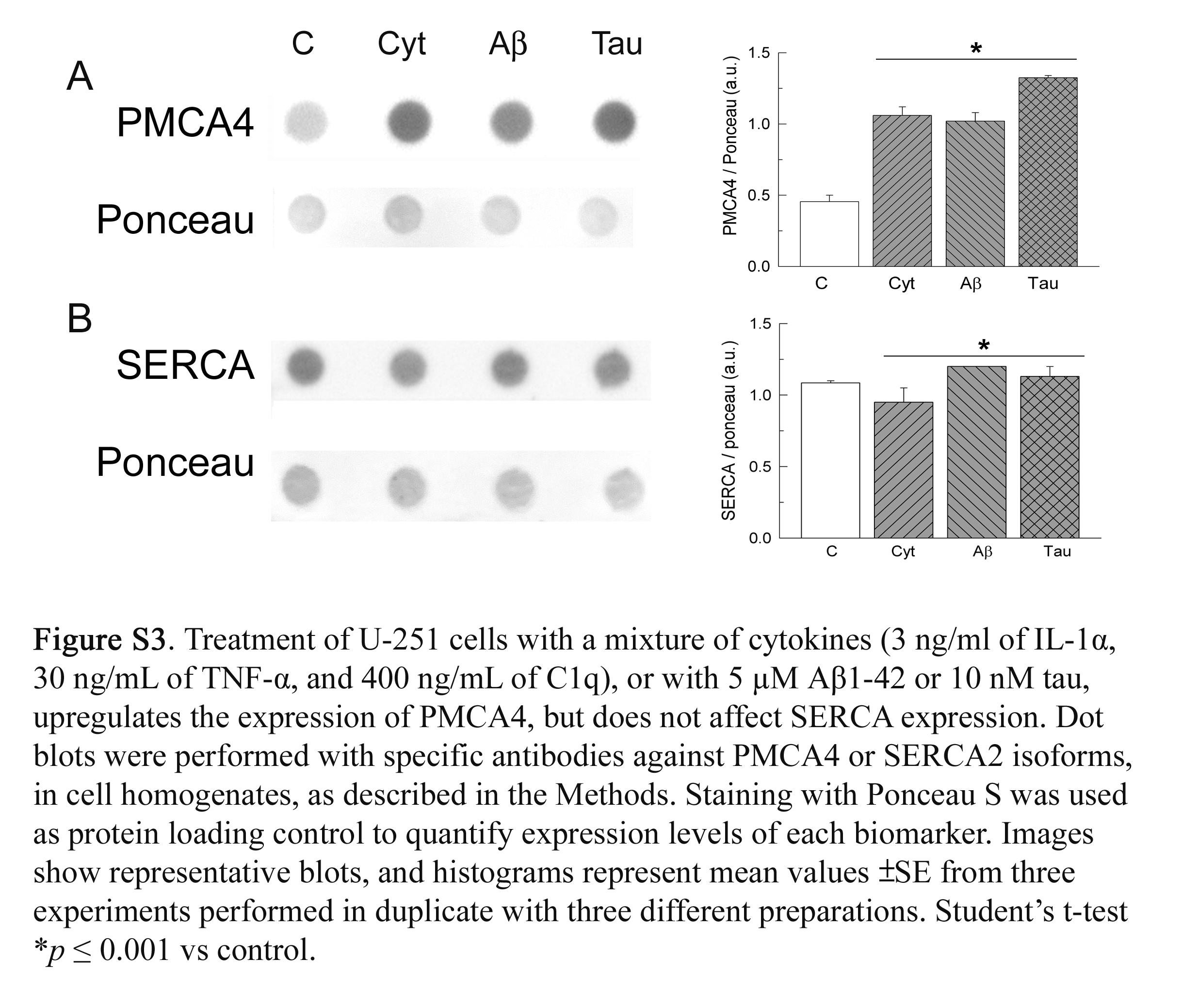

Supplement: Supplementary file 3 — Fig. S3. Treatment of U‐251 cells with a mixture of cytokines (3 ng·mL−1 of IL‐1α, 30 ng·mL−1 of TNF‐α, and 400 ng·mL−1 of C1q), or with 5 μm Aβ1‐42 or 10 nm tau, upregulates the expression of PMCA4, but does not affect SERCA expression. [file FEB4-15-1350-s004.tif]
