# Supplementary material for: Neurotoxic amyloid β‐peptide and tau produce cytokine‐like effects on PMCA in glioblastoma cell lines, enhancing its activity and isoforms expression
Source: FEBS Open Bio. 2025 May 5;15(8):1350–64. doi: 10.1002/2211-5463.70046 (PMC12319713; doi:10.1002/2211-5463.70046)
Supplement: Supplementary file 4 — Table S1. Downregulation of Ca2+‐ATPase activity in neuronal cell lines, after been treated with cytokines (3 ng·mL−1 of IL‐1α, 30 ng·mL−1 of TNF‐α, and 400 ng·mL−1 of C1q), or with 5 μm Aβ1‐42 or 10 nm tau. [file FEB4-15-1350-s002.docx]

| **Table S1.** Downregulation of Ca^2+^-ATPase activity in neuronal cell lines, after been treated with cytokines (3 ng/ml of IL-1α, 30 ng/mL of TNF-α, and 400 ng/mL of C1q), or with 5 µM Aβ1-42 or 10 nM tau. Values represent mean ± SE from three experiments performed in duplicate and with three different preparations. *P* values were obtained using Student’s t-test. | | | |
| --- | --- | --- | --- |
| **Cell line** | **Treatment** | **Activity**  **(µmol. min^-1^.mg^-1^)** | ***P* value** |
| SH-SY5Y | Control  Cytokines  Aβ  Tau | 0.292 ± 0.01  0.180 ± 0.0005  0.189 ± 0.006  0.191 ± 0.001 | 0.009  0.01  0.01 |
| HT-22 | Control  Cytokines  Aβ  Tau | 0.430 ± 0.001  0.298 ± 0.01  0.228 ± 0.02  0.250 ± 0.01 | 0.0004  0.0006  0.0004 |
| N2a | Control  Cytokines  Aβ  Tau | 0.315 ± 0.005  0.187 ± 0.006  0.175 ± 0.007  0.180 ± 0.004 | 0.0004  0.0001  0.0004 |
